# 6‐Aminoflavone Inhibits Planktonic Growth, Disrupts Biofilm Architecture, and Regulates Quorum Sensing in *Klebsiella pneumoniae*

**DOI:** 10.1155/bri/8058282

**Published:** 2026-08-21

**Authors:** Pooja Pandey, T. Tushar, Lakshmi Sirisha Vavilala

**Affiliations:** ^1^ School of Biological Sciences, UM DAE Centre for Excellence in Basic Sciences, University of Mumbai, Vidyanagari Kalina Campus, Mumbai 400098, India, mu.ac.in

**Keywords:** 6-Aminoflavone, apoptosis, biofilm disruption, planktonic growth inhibition, quorum sensing

## Abstract

Antibiotic resistance has emerged as a major global health challenge, particularly in biofilm‐forming pathogens that exhibit enhanced tolerance to antimicrobial therapies. *K*. *pneumoniae*, a multidrug‐resistant pathogen, is a leading cause of hospital‐acquired infections, including pneumonia, septicemia, and device‐associated infections. Its robust biofilm‐forming capacity facilitates immune evasion, restricts antibiotic penetration, and contributes to recurrent infections. Therefore, the identification of agents capable of targeting both planktonic bacterial growth and biofilm architecture is of considerable therapeutic importance. In the present study, the antibacterial, antibiofilm, and antivirulence potential of 6‐Aminoflavone was investigated against *K. pneumoniae*. Antibacterial activity was evaluated using growth inhibition assays, time–kill kinetics, and clonogenic survival analysis. Mechanistic investigations revealed significant intracellular ROS accumulation, increased oxidative stress susceptibility, and induction of apoptosis‐like cell death, suggesting ROS‐mediated antibacterial activity. In addition to suppressing bacterial proliferation, 6‐Aminoflavone exhibited promising antibiofilm efficacy, inhibiting biofilm formation by 86.23% and eradicating 84.77% of established biofilms. These effects were associated with a substantial reduction in cell surface hydrophobicity (∼45.39%), and EPS levels were reduced to ∼2.33% as compared to untreated controls, indicating severe destabilization of the biofilm matrix. Furthermore, eDNA, a key structural component of the biofilm scaffold, exhibited a maximum ∼2.13‐fold reduction following treatment. Confocal microscopy confirmed marked disruption and collapse of biofilm architecture. Additionally, quorum sensing (QS)–regulated virulence factors, including urease (∼51.92%), protease (∼55.18%), and lipase (∼44.90%), were significantly attenuated. These findings demonstrate that 6‐Aminoflavone exerts antimicrobial activity by inducing oxidative stress–mediated apoptosis‐like bacterial death, disrupting biofilm structural components, and suppressing QS‐regulated virulence in *K. pneumoniae*.

## 1. Introduction

Antibiotic resistance represents a profound global health crisis, currently responsible for over 1.27 million direct deaths annually and nearly 5 million deaths associated worldwide, with projections estimating up to 10 million deaths per year by 2050 if unchecked [1]. Among the major contributors to antimicrobial failure is bacterial biofilm formation, a highly organized survival strategy that confers remarkable tolerance to antibiotics and host immune responses without requiring genetic resistance mutations [2]. Biofilm‐embedded cells exhibit metabolic heterogeneity, restricted antimicrobial penetration, activation of stress pathways, and enhanced horizontal gene transfer, collectively promoting persistence and therapeutic failure [2].

*K*. *pneumoniae* is a Gram‐negative opportunistic pathogen categorized by the World Health Organization (WHO) as a critical‐priority “superbug” due to its escalating multidrug resistance, including extended‐spectrum β‐lactamase (ESBL) and carbapenem‐resistant strains [3]. Carbapenem‐resistant *K. pneumoniae* infections are associated with mortality rates exceeding 40% in invasive cases [4]. Clinically, it is a predominant cause of ventilator‐associated pneumonia, catheter‐associated urinary tract infections, bloodstream infections, and sepsis, particularly in hospitalized and immunocompromised patients [5]. A defining feature underlying its clinical persistence is its robust biofilm‐forming capacity on both biotic and abiotic surfaces, including indwelling medical devices [6].

Biofilm formation in *K. pneumoniae* is mediated by type 1 and type 3 fimbriae, capsular polysaccharides, lipopolysaccharides, and outer membrane proteins, which coordinate adhesion and matrix development [7]. Importantly, quorum sensing (QS) signaling systems regulate collective gene expression, controlling extracellular polymeric substance (EPS) synthesis, virulence factor production, stress adaptation, and community behavior [8]. Disrupting QS pathways represents a promising strategy to attenuate biofilm maturation without exerting strong bactericidal pressure, thereby reducing the likelihood of resistance development [9].

Given the toxicity and diminishing efficacy of conventional antibiotics, natural bioactive compounds with minimal adverse effects are increasingly being explored as antibiofilm and antivirulence agents. Flavonoids, a diverse class of plant‐derived polyphenols, possess antimicrobial, antioxidant, anti‐inflammatory, and QS inhibitory activities [10, 11]. Mechanistically, flavonoids can induce oxidative stress, alter membrane permeability, suppress virulence gene expression, and inhibit EPS production [11, 12].

6‐Aminoflavone, an amino‐substituted flavone derivative, exhibits enhanced biological activity compared to its parent scaffold due to improved redox reactivity and cellular interaction [13]. While emerging evidence supports its antibiofilm potential in certain Gram‐negative pathogens [14, 15], its mechanistic impact on biofilm formation and QS regulation in *K*. *pneumoniae* remains largely unexplored. Therefore, the present study aims to systematically evaluate the antibacterial and antibiofilm activity of 6‐Aminoflavone against *K. pneumoniae* and elucidate its effects on oxidative stress induction, EPS modulation, and biofilm structural integrity.

## 2. Materials and Methods

### 2.1. Bacterial Strain and Compound Preparation

*K*. *pneumoniae* strain (MTCC 432) was procured from the Microbial Type Culture Collection and Gene Bank (MTCC), Chandigarh, India. The strain was authenticated by the supplier based on standard genotypic and phenotypic characterization, including 16S rRNA gene sequence analysis and biochemical profiling. *K*. *pneumoniae* was cultured in growth medium 3 (GM3) under aerobic conditions. 6‐Aminoflavone was procured from Sigma‐Aldrich, USA (catalog no. 576263), with a stated purity of 97% (w/w) as provided by the manufacturer. The compound was dissolved in absolute ethanol to prepare a stock solution (5 mg/mL) and subsequently diluted to the required working concentrations (0–1000 μg/mL). The final ethanol concentration in all assays did not exceed 1% (v/v). Corresponding ethanol‐only controls were included in all experiments to exclude solvent‐associated effects, while untreated bacterial cultures served as experimental controls.

#### 2.1.1. Bacterial Culture Conditions

*K*. *pneumoniae* was routinely maintained on GM3 agar plates (2% agar) at 37°C and periodically subcultured to ensure viability. For experimental assays, a single isolated colony was inoculated into GM3 broth and incubated aerobically at 37°C overnight, after which the cultures were diluted to the required inoculum density for individual experiments.

### 2.2. Minimum Inhibitory Concentration (MIC) and Minimum Bactericidal Concentration (MBC)

The MIC of 6‐Aminoflavone was determined using the modified broth microdilution method in accordance with Clinical and Laboratory Standards Institute (CLSI) guidelines coupled with 3‐(4,5‐dimethylthiazol‐2‐yl)‐2,5‐diphenyltetrazolium bromide (MTT)–based metabolic viability assessment. Briefly, bacterial suspensions were adjusted to ∼1 × 10^6^ colony‐forming unit (CFU)/mL and dispensed into sterile 96‐well microtiter plates containing serial two‐fold dilutions of 6‐Aminoflavone (0–1000 μg/mL). Plates were incubated at 37°C for 24 h, 120 rpm. Following incubation, bacterial viability was assessed using the MTT assay according to standard protocols. Appropriate media controls, untreated bacterial controls, and solvent controls were included in all experiments to eliminate background interference and minimize false‐positive observations. Absorbance was measured at 570 nm, and the MIC was defined as the lowest concentration of 6‐Aminoflavone that produced complete inhibition of bacterial viability relative to the untreated control [16], [17–19].

For determination of the MBC, aliquots (100 μL) from wells showing no visible growth were plated onto GM3 agar and incubated at 37°C for 24 h. The MBC was defined as the lowest concentration of 6‐Aminoflavone that resulted in no observable colony formation, indicating ≥ 99.9% reduction in viable bacterial counts. All experiments were performed in triplicate to ensure reproducibility [17–19].

### 2.3. Time–Kill Assay and CFU Assay

*K*. *pneumoniae* cells (∼1 × 10^8^ CFU/mL) were exposed to increasing concentrations of 6‐Aminoflavone (0–1000 μg/mL) and incubated at 37°C, 100 rpm for 24 h. To monitor growth kinetics, aliquots (100 μL) were collected from treated and untreated cultures at defined time intervals and plated onto agar plates. After incubation, colony counts were determined, and bacterial growth was expressed as log_10_ CFU/mL over time to generate time–kill curves [19].

For assessment of clonogenic survival, bacterial cultures were treated with varying concentrations of 6‐Aminoflavone for 24 h under identical conditions. Following treatment, samples were serially diluted to approximately 1 × 10^6^ CFU/mL, and 100 μL of the appropriate dilution was spread onto GM3 agar plates. Plates were incubated at 37°C for 24 h, after which CFUs were enumerated. Viability was calculated relative to untreated controls [20].

### 2.4. Intracellular Reactive Oxygen Species (ROS) Determination and Hydrogen Peroxide Sensitivity Test

Intracellular ROS generation induced by 6‐Aminoflavone was quantified using the fluorescent probe 2′,7′‐dichlorodihydrofluorescein diacetate (DCFDA). This cell‐permeable dye is hydrolyzed by intracellular esterases to a nonfluorescent intermediate, which is subsequently oxidized by ROS to yield a fluorescent product (DCF), thereby enabling detection of oxidative stress. *K*. *pneumoniae* cultures were grown to mid‐log phase (OD_600_ ≈ 0.5) and exposed to increasing concentrations of 6‐Aminoflavone (0–1000 μg/mL) in the presence of DCFDA. Fluorescence intensity was monitored at excitation/emission wavelengths of 485/535 nm using a microplate reader. Measurements were recorded at hourly intervals up to 5 h, and ROS production was expressed relative to untreated controls [17, 21].

To further evaluate oxidative stress susceptibility, a hydrogen peroxide (H_2_O_2_) sensitivity assay was performed. Bacterial cultures were treated with varying concentrations of 6‐Aminoflavone for 24 h, followed by dilution to approximately 1 × 10^3^ CFU/mL. Aliquots (1 mL) of the diluted suspension were mixed with 19 mL of molten 2% LB agar and poured into sterile plates. After solidification, sterile 6‐mm Whatman filter paper discs were placed on the agar surface and loaded with 10 μL of 3% H_2_O_2_. Plates were incubated at 37°C for 24 h, and zones of inhibition surrounding the discs were measured. Increased zone diameters were interpreted as enhanced susceptibility to oxidative stress [22].

### 2.5. Detection of Apoptosis‐Like Cell Death Induced by 6‐Aminoflavone in *K*. *pneumoniae* Using Annexin V–FITC/Propidium Iodide (PI) Assay

The ability of 6‐Aminoflavone to induce apoptosis‐like cell death in *K*. *pneumoniae* was investigated using an Annexin V–FITC and PI dual staining apoptosis detection kit (MedChemExpress, USA) coupled with flow cytometric analysis. This approach enables differentiation of bacterial populations based on membrane asymmetry and permeability, distinguishing viable, early apoptosis‐like, late apoptosis‐like, and necrotic cells. Briefly, overnight cultures of *K. pneumoniae* were exposed to increasing concentrations of 6‐Aminoflavone and incubated at 37°C for 24 h, 120 rpm. Untreated cultures were maintained as controls. Following treatment, cells were harvested by centrifugation, washed twice with sterile phosphate‐buffered saline (PBS), and resuspended in Annexin V binding buffer as per the manufacturer’s instructions. Cells were stained with Annexin V–FITC to detect externalization of phosphatidylserine (PS), a characteristic feature of apoptosis‐like membrane alterations. After incubation in the dark at room temperature, PI was added to assess membrane integrity and identify cells with compromised membranes. Samples were gently mixed and immediately subjected to flow cytometric analysis. Data acquisition was performed using a BD FACSVerse flow cytometer, with a minimum of 30,000 events recorded per sample. Appropriate controls, including unstained, single‐stained, and compensation controls, were included to ensure accurate gating and fluorescence compensation. Cell populations were classified as viable (Annexin V–negative/PI–negative), early apoptosis‐like (Annexin V–positive/PI–negative), late apoptosis‐like (Annexin V–positive/PI–positive), and necrotic (Annexin V–negative/PI–positive). Data were analyzed using BD FACSuite software [23].

### 2.6. Biofilm Inhibition Assay

The antibiofilm activity of 6‐Aminoflavone against *K. pneumoniae* was evaluated using the crystal violet microtiter plate assay. Bacterial cultures prepared as described under “Bacterial Culture Conditions” were exposed to increasing concentrations of 6‐Aminoflavone (0–1000 μg/mL) and incubated at 37°C for 24 h to allow biofilm formation. Following incubation, nonadherent cells were removed, and the attached biofilm biomass was quantified by crystal violet staining. The retained dye was solubilized in absolute ethanol, and absorbance was measured at 595 nm. Biofilm inhibition was calculated as the percentage reduction in biofilm biomass relative to untreated controls. This assay was employed to determine the ability of 6‐Aminoflavone to prevent biofilm establishment by *K. pneumoniae* under *in vitro* conditions [24–27].

### 2.7. Cell Surface Hydrophobicity (CSH) Assay

CSH of *K*. *pneumoniae* was evaluated using the microbial adhesion to hydrocarbons (MATH) assay. Briefly, bacterial cultures prepared as described under “Bacterial Culture Conditions” were treated with increasing concentrations of 6‐Aminoflavone (0–1000 μg/mL). Following treatment, the initial absorbance of the bacterial suspension was recorded at 600 nm (A_0_), after which 1 mL of toluene was added and samples were vortexed to facilitate bacterial adhesion to the hydrocarbon phase. Following phase separation, the absorbance of the aqueous phase was measured at 600 nm (A_1_). CSH was calculated using the following formula. All experiments were performed in triplicate [28, 29].

(1) CSH %=A0−A1A0×100.

### 2.8. Biofilm Eradication Assay

The biofilm eradication potential of 6‐Aminoflavone against established *K*. *pneumoniae* biofilms was evaluated using a crystal violet–based microtiter plate assay. Bacterial cultures prepared as described under “Bacterial Culture Conditions” were supplemented with hydrogen peroxide (H_2_O_2_) to accelerate biofilm maturation through oxidative stress–mediated biofilm induction and matrix production [30]. Aliquots were incubated in 96‐well plates at 37°C for 24 h to generate mature biofilms. Following removal of planktonic cells, preformed biofilms were exposed to increasing concentrations of 6‐Aminoflavone (0–1000 μg/mL) for an additional 24 h. Residual biofilm biomass was quantified by crystal violet staining and absorbance measurement at 595 nm. Biofilm eradication was expressed as the percentage reduction in biomass relative to untreated biofilm controls [30, 31].

### 2.9. Quantification of EPS Production

The effect of 6‐Aminoflavone on EPS production by *K. pneumoniae* was evaluated using a gravimetric quantification assay. Bacterial cultures prepared as described under “Bacterial Culture Conditions” were supplemented with hydrogen peroxide (H_2_O_2_; 17 μL per 10 mL culture) to promote oxidative stress–induced biofilm maturation and EPS synthesis. Following incubation for 24 h, cultures were exposed to increasing concentrations of 6‐Aminoflavone (0–1000 μg/mL) and incubated at 37°C, 120 rpm for an additional 24 h. Cell‐free supernatants were collected by centrifugation, and EPS was precipitated using 10% (w/v) trichloroacetic acid (TCA) followed by chilled acetone. The recovered EPS pellet was dried to constant weight and quantified gravimetrically. EPS inhibition was calculated relative to untreated controls to determine the ability of 6‐Aminoflavone to suppress matrix production associated with biofilm development [32].

### 2.10. Quantification of Extracellular DNA (eDNA) Degradation in Mature Biofilms

The effect of 6‐Aminoflavone on eDNA, a key structural and functional component of the biofilm EPS matrix, was investigated in preestablished *K*. *pneumoniae* biofilms. Mature biofilms were generated and treated as described above, followed by eDNA extraction from the biofilm matrix. Briefly, biofilm samples were subjected to EDTA‐mediated matrix destabilization, and eDNA was purified through sequential phenol–chloroform extraction and ethanol precipitation. The recovered DNA was resuspended in TE buffer, and eDNA quantity and purity were determined spectrophotometrically at 260 nm. Reduction in eDNA levels following treatment was used as an indicator of biofilm matrix disruption and impaired structural integrity, thereby assessing the ability of 6‐Aminoflavone to target a critical scaffold component involved in biofilm stability and persistence [32, 33].

### 2.11. Confocal Laser Scanning Microscopy (CLSM) Analysis of Biofilm Architecture

Biofilm architecture and extracellular matrix composition of *K*. *pneumoniae* were analyzed using CLSM. Biofilms were developed on sterile glass coverslips under static conditions and subsequently exposed to subinhibitory (½× MIC) and inhibitory (MIC) concentrations of 6‐Aminoflavone for 24 h at 37°C. Untreated biofilms were included as controls.

Following treatment, coverslips were gently washed twice with sterile PBS to remove planktonic and weakly attached cells. Biofilms were then stained to visualize major extracellular matrix components. Exopolysaccharides were labeled using TRITC‐conjugated Concanavalin A (ConA) (100 μg/mL), whereas eDNA was stained with PI. All staining steps were carried out in the dark to minimize photobleaching. After staining, excess dye was removed by rinsing with nuclease‐free water, and coverslips were air‐dried and mounted onto glass slides. Imaging was performed using a Zeiss LSM 980 confocal microscope equipped with a 63× oil‐immersion objective. TRITC‐ConA was excited at 488 nm, and PI was excited at 563 nm. Emission signals were collected within the ranges of 493–561 nm and 565–727 nm for ConA and PI, respectively. Z‐stack images were acquired at 0.125 μm intervals throughout the biofilm thickness to enable three‐dimensional reconstruction. Quantitative image analysis was carried out using Zeiss ZEN software. Fluorescence intensity and structural parameters were calculated from two independent regions per biofilm across three biological replicates [29, 34–36].

### 2.12. Quantification of QS‐Regulated Virulence Factor Production in *K*. *pneumoniae*

The impact of 6‐Aminoflavone on QS‐associated virulence in *K*. *pneumoniae* was examined by measuring QS‐induced virulence factors production. Overnight cultures were exposed to increasing concentrations of 6‐Aminoflavone and incubated at 37°C for 24 h, 120 rpm. Untreated cultures were maintained as controls. To ensure that the observed changes in enzyme activity were not attributable to differences in bacterial cell density, cultures were normalized to ∼1 × 10^8^ CFU/mL following 6‐Aminoflavone treatment before enzyme assays, enabling assessment of 6‐Aminoflavone’s specific effects on virulence factors production.

#### 2.12.1. Lipase Activity Assay

Following treatment, bacterial cells were collected by centrifugation and resuspended in 500 μL of Tris–EDTA buffer (pH 8.0). Cell lysis was achieved via sonication, and the lysates were subsequently centrifuged to obtain cell‐free supernatants. Lipase activity was determined by incubating the supernatant with *p*‐nitrophenyl palmitate (*p*‐NPP) as the substrate at 60°C for 20 min. Enzymatic hydrolysis of *p*‐NPP results in the release of *p*‐nitrophenol, which was quantified spectrophotometrically by measuring absorbance at 410 nm [37].

#### 2.12.2. Urease Activity Assay

Urease activity was determined using cell‐free supernatants obtained from treated and untreated *K*. *pneumoniae* cultures following centrifugation. The supernatant was incubated with a urea‐containing substrate at 37°C for 3 h to allow enzymatic hydrolysis. After incubation, Nessler’s reagent was added to detect ammonia released during urea degradation. The resulting colorimetric change was measured spectrophotometrically at 530 nm, and urease activity was quantified relative to untreated controls [38].

#### 2.12.3. Protease Activity Assay

Protease activity was evaluated using an azocasein‐based assay. Culture supernatants were incubated with azocasein prepared in Tris–HCl buffer under appropriate conditions. The reaction was terminated by the addition of TCA to precipitate undigested proteins. Samples were subsequently cooled and centrifuged to remove precipitates, and the absorbance of the supernatant was measured after the addition of NaOH. Proteolytic activity was quantified spectrophotometrically at 400 nm. All assays were conducted in triplicate, and enzyme activities were expressed relative to untreated controls [39].

### 2.13. Statistical Analysis

All experiments were performed in triplicate, and data are presented as mean ± standard deviation (SD). Statistical analyses were carried out using OriginPro 8.5 software. Differences between groups were evaluated using one‐way analysis of variance (ANOVA), followed by Turkey’s post hoc test for multiple comparisons. A *p* value < 0.05 was considered statistically significant.

## 3. Results

### 3.1. MIC and MBC

The antimicrobial activity of 6‐Aminoflavone against *K*. *pneumoniae* was evaluated using MIC and MBC assays. A concentration‐dependent inhibition of bacterial growth was observed, with an MIC value of 620 μg/mL. At this concentration, metabolic activity was significantly reduced relative to untreated controls, indicating effective suppression of bacterial proliferation. Assessment of bactericidal activity revealed no substantial reduction in viable counts at concentrations corresponding to the MIC, suggesting that 6‐Aminoflavone primarily exerts a bacteriostatic effect under the tested conditions. The MBC values were notably higher than the MIC, further supporting growth inhibition rather than rapid bacteriolysis as the dominant mode of action. These results demonstrate that 6‐Aminoflavone effectively impairs *K. pneumoniae* growth in a dose‐dependent manner, predominantly through a bacteriostatic mechanism.

### 3.2. 6‐Aminoflavone Induces Concentration‐ and Time‐Dependent Growth Suppression and Loss of Colony‐Forming Ability in *K*. *pneumoniae*

**FIGURE 1:**
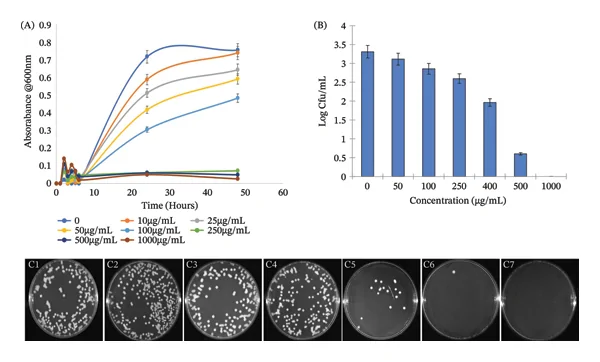
Antibacterial activity of 6‐Aminoflavone against *Klebsiella pneumoniae*. (A) Time–kill kinetic**s** of *K. pneumoniae* in the presence of increasing concentrations of 6‐Aminoflavone over a 48‐h incubation period, demonstrating concentration‐dependent suppression of bacterial growth**.** (B) Quantitative analysis of viable cells**,** expressed as colony‐forming units (CFUs), shows a progressive decline in bacterial viability following treatment. (C1–C7) Representative CFU plates illustrating the gradual reduction in colony formation with increasing concentrations of 6‐Aminoflavone, confirming its effective antibacterial effect**.** Data represent the mean ± SD of three independent experiments**,** and statistical significance was considered at *p*  <  0.05.

The antimicrobial activity of 6‐Aminoflavone against *K*. *pneumoniae* was further evaluated using a time‐dependent growth–kill assay over 48 h, complemented by clonogenic survival analysis. Untreated cultures displayed a typical bacterial growth curve with a pronounced exponential phase, reflecting viable bacterial cell counts and proliferation over time. In contrast, exposure to 6‐Aminoflavone resulted in a marked, concentration‐dependent decline in growth kinetics. Moderate concentrations (25–100 μg/mL) produced a noticeable reduction in culture density, indicating partial inhibition of bacterial proliferation. At higher concentrations (500–1000 μg/mL), the growth curve remained close to baseline throughout the incubation period, indicating near‐complete suppression of bacterial expansion. Importantly, the exponential growth phase was attenuated, suggesting a substantial inhibition of viable bacterial cell counts and proliferation over time and indicating a pronounced bacteriostatic effect of 6‐Aminoflavone (Figure 1A).

Clonogenic survival assays further corroborated these observations, revealing a clear dose‐dependent reduction in viable colony formation following 6‐Aminoflavone treatment. While untreated cultures produced dense and confluent colonies, treatment with 6‐Aminoflavone at 50–250 μg/mL resulted in a noticeable decline in colony numbers. A pronounced suppression of clonogenic survival was evident at 400 μg/mL, with only sparse colonies detectable near 500 μg/mL and complete inhibition of colony formation at 1000 μg/mL. Quantitative enumeration (log CFU/mL) further confirmed a progressive decline in bacterial viability with increasing concentrations of 6‐Aminoflavone (Figure 1B, C1–C7). Collectively, these findings demonstrate that treatment with 6‐Aminoflavone exerts concentration‐dependent antimicrobial effects, markedly impairing the clonogenic potential of *K. pneumoniae*, particularly at concentrations approaching and exceeding the MIC.

### 3.3. Quantification of Intracellular ROS Induction and Oxidative Stress Sensitization by 6‐Aminoflavone in *K*. *pneumoniae*

**FIGURE 2:**
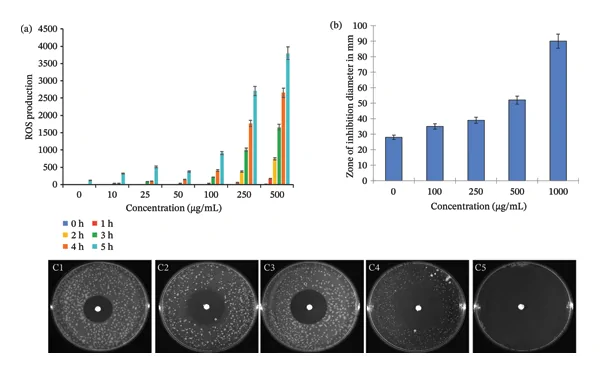
Oxidative stress induction in *Klebsiella pneumoniae* following treatment with 6‐Aminoflavone. (A) Intracellular reactive oxygen species (ROS) generation was measured after exposure to 6‐Aminoflavone, demonstrating a concentration‐dependent increase in oxidative stress. (B) Hydrogen peroxide (H_2_O_2_) sensitivity assay showing increased susceptibility of treated cells to oxidative damage. (C1–C5) Representative agar plates displaying enlarged inhibition zones around H_2_O_2_ discs following treatment, indicating enhanced oxidative stress–mediated growth inhibition. Values represent mean ± SD from three independent experiments, and statistical significance was considered at *p*  <  0.05.

Intracellular ROS generation was quantified to determine whether 6‐Aminoflavone induces oxidative stress in *K*. *pneumoniae*. Treatment with 6‐Aminoflavone produced a pronounced concentration‐ and time‐dependent increase in ROS accumulation, indicating progressive disruption of bacterial redox homeostasis. While lower concentrations (10–50 μg/mL) elicited minimal changes comparable to untreated controls, exposure to 250 μg/mL resulted in a marked elevation in ROS levels, increasing from baseline at 0 h to ∼12.35‐fold at 3 h, 20.52‐fold at 4 h, and 32.01‐fold at 5 h. A more robust oxidative response was observed at 500 μg/mL, where ROS levels rose to ∼19.10‐fold at 3 h, 30.81‐fold at 4 h, and 43.40‐fold at 5 h relative to the initial measurement (Figure 2A). These findings demonstrate that 6‐Aminoflavone induces a strong oxidative burst in *K. pneumoniae*, highlighting redox imbalance as a key mechanism underlying its antimicrobial activity.

To further evaluate oxidative stress susceptibility, hydrogen peroxide (H_2_O_2_) sensitivity assays were performed in *K*. *pneumoniae* following treatment with 6‐Aminoflavone. A concentration‐dependent increase in the inhibition zone diameter was observed, indicating enhanced vulnerability of the bacteria to oxidative stress. Compared with the untreated control (28 mm), the inhibition zone progressively increased to 35 mm at 100 μg/mL, 39 mm at 250 μg/mL, 52 mm at 500 μg/mL, and reached a maximum of 90 mm at 1000 μg/mL (Figure 2B). The marked enlargement of inhibition zones at higher concentrations demonstrates a substantial increase in oxidative stress sensitivity (Figure 2C1–C5). Together with the observed intracellular ROS accumulation, these results indicate that 6‐Aminoflavone potentiates oxidative stress and weakens bacterial redox defense systems. Collectively, these findings suggest that disruption of redox homeostasis represents a key mechanism underlying the antimicrobial activity of 6‐Aminoflavone against *K*. *pneumoniae.*

### 3.4. 6‐Aminoflavone Induces Apoptosis‐Like Cell Death in *K*. *pneumoniae*

**FIGURE 3:**
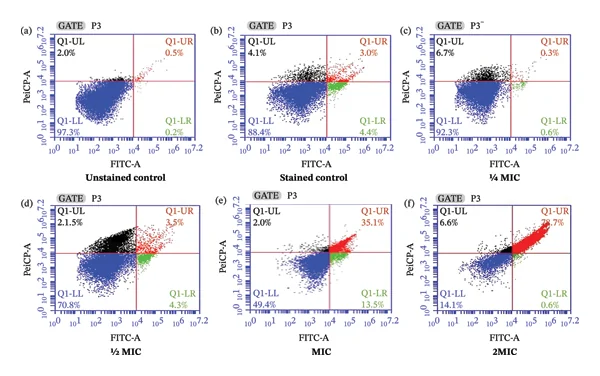
Detection of apoptosis‐like cell death in *Klebsiella pneumoniae* following treatment with 6‐Aminoflavone using Annexin V–FITC/propidium iodide (PI) staining. Representative flow cytometry dot plots showing phosphatidylserine externalization and membrane integrity: (A) unstained control, (B) stained control, (C) 1/4 MIC, (D) 1/2 MIC, (E) MIC, and (F) 2× MIC of 6‐Aminoflavone. Quadrants represent viable (Annexin V^−^/PI^−^), early apoptosis‐like (Annexin V^+^/PI^−^), late apoptosis‐like (Annexin V^+^/PI^+^), and membrane‐compromised/dead (Annexin V^−^/PI^+^) cell populations. A concentration‐dependent increase in Annexin V–positive populations indicates progressive induction of apoptosis‐like cell death. Data are presented as mean ± SD of three independent experiments (*p*  <  0.05).

To elucidate the mode of cell death induced by 6‐Aminoflavone, *K*. *pneumoniae* cells were subjected to Annexin V–FITC/PI dual staining followed by flow cytometric analysis, which enables discrimination of viable, early apoptosis‐like, late apoptosis‐like, and necrotic populations based on PS externalization and membrane integrity (Figure 3). In the unstained control, the bacterial population was predominantly viable (97.3%), with only minor fractions corresponding to early apoptosis‐like (0.2%), late apoptosis‐like (0.5%), and necrotic (2%) cells, confirming minimal background staining. In the stained untreated control, the majority of cells remained viable (88.4%), with smaller populations representing early apoptosis‐like (4.4%), late apoptosis‐like (3%), and necrotic (4.1%) cells (Figure 3A). Following 6‐Aminoflavone treatment, a concentration‐dependent redistribution of cell populations was observed. At 1/4 MIC, most cells remained viable (92.3%), while 0.6% exhibited early apoptosis‐like features, 0.3% late apoptosis‐like features, and 6.7% were necrotic (Figure 3B). At 1/2 MIC, viability declined to 70.8%, accompanied by increases in early apoptosis‐like (4.3%), late apoptosis‐like (3.5%), and necrotic (21.5%) populations (Figure 3C). Treatment at the MIC resulted in a pronounced redistribution of cell populations, with 49.4% viable cells, 13.5% early apoptosis‐like cells, 35.1% late apoptosis‐like cells, and 2% necrotic cells (Figure 3D). At the highest concentration, 2×MIC, cell viability dropped sharply to 14.1%, while the majority of cells transitioned into late apoptosis‐like death (78.7%), with 0.6% early apoptosis‐like and 6.6% necrotic populations (Figure 3E). Overall, increasing concentrations of 6‐Aminoflavone resulted in a progressive shift from viable cells toward apoptosis‐like populations, with late apoptosis‐like cell death predominating at MIC and 2×MIC levels. This pattern indicates that 6‐Aminoflavone primarily induces apoptosis‐like cell death rather than immediate necrotic lysis in *K. pneumoniae*. Together with the observed ROS accumulation and loss of viability, these findings support a mechanism involving oxidative stress–mediated disruption of membrane asymmetry, progressive membrane damage, and eventual loss of bacterial viability.

### 3.5. 6‐Aminoflavone Inhibits Biofilm Establishment and Attenuates CSH in *K*. *pneumoniae*

**FIGURE 4:**
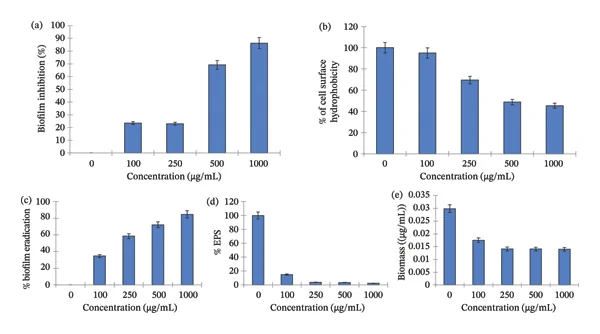
Antibiofilm effects of 6‐Aminoflavone against *Klebsiella pneumoniae*. (A) Inhibition of biofilm formation, (B) assessment of cell surface hydrophobicity, (C) eradication of preestablished biofilms, (D) quantitative analysis of extracellular polymeric substances (EPS), and (E) quantification of extracellular DNA (eDNA) within the biofilm matrix. Results are presented as mean ± SD from three independent experiments (*p*  <  0.05).

The antibiofilm activity of 6‐Aminoflavone against *K*. *pneumoniae* was evaluated using the crystal violet assay to quantify total biofilm biomass. Treatment with 6‐Aminoflavone produced a clear concentration‐dependent inhibition of biofilm formation across the tested range (100–1000 μg/mL). Overall, biofilm biomass was reduced from ∼23% inhibition at lower concentrations to a maximum of 86.23% inhibition at 1000 μg/mL compared with the untreated control (Figure 4A). These findings demonstrate that 6‐Aminoflavone effectively disrupts biofilm development in a dose‐dependent manner, highlighting its antibiofilm potential against *K. pneumoniae.*

To determine whether the observed biofilm inhibition was associated with changes in bacterial surface properties, CSH was evaluated using the MATH assay. Untreated *K*. *pneumoniae* cells exhibited high surface hydrophobicity (∼100%), whereas treatment with 6‐Aminoflavone caused a progressive, concentration‐dependent decline in CSH. Exposure to 100 μg/mL produced a modest reduction (95.07%), while a more pronounced decrease was observed at 250 μg/mL, where hydrophobicity declined to 69.55%. At higher concentrations, CSH decreased substantially, reaching 48.76% at 500 μg/mL and 45.39% at 1000 μg/mL (Figure 4B). The parallel reduction in biofilm biomass and CSH indicates that 6‐Aminoflavone disrupts bacterial surface physicochemical properties, thereby impairing initial adhesion and surface‐associated interactions essential for biofilm establishment. These findings suggest that modulation of CSH represents a key mechanistic contributor to the antibiofilm activity of 6‐Aminoflavone against *K. pneumoniae*.

### 3.6. 6‐Aminoflavone Disrupts Biofilm Architecture by Destabilizing EPS Matrix Integrity in *K*. *pneumoniae*

The biofilm‐disruptive activity of 6‐Aminoflavone against pre‐formed *K*. *pneumoniae* biofilms was evaluated using a biofilm eradication assay. Treatment of mature biofilms resulted in a clear concentration‐dependent reduction in biofilm biomass (Figure 4C). Biofilm eradication increased progressively with increasing concentrations, reaching 34.62% at 100 μg/mL and 58.44% at 250 μg/mL, while higher concentrations produced more pronounced effects with 72.15% and 84.78% eradication at 500 and 1000 μg/mL, respectively (Figure 4C). These findings demonstrate that 6‐Aminoflavone effectively disrupts established biofilms, which are typically highly tolerant to antimicrobial treatment. Analysis of EPS revealed a dramatic and progressive depletion following 6‐Aminoflavone treatment. Relative to untreated controls (100%), EPS levels declined sharply to 14.87% at 100 μg/mL (∼6.72‐fold reduction) and further decreased to 3.80%, 3.49%, and 2.33% at 250, 500, and 1000 μg/mL, corresponding to approximately 26.33‐, 28.65‐, and 42.86‐fold reductions, respectively (Figure 4D). This substantial loss of EPS indicates severe destabilization of the biofilm matrix, which is critical for maintaining biofilm cohesion, structural integrity, and protective capacity.

To further characterize matrix destabilization, eDNA—a key structural and signaling component of mature biofilms—was quantified. Biofilms established under oxidative stress conditions and subsequently treated with 6‐Aminoflavone showed a progressive decline in eDNA levels. Compared with the untreated control (0.0298), eDNA concentrations decreased to 0.0175 at 100 μg/mL (∼1.70‐fold reduction) and further to 0.0141, 0.01408, and 0.0139 at 250, 500, and 1000 μg/mL, representing approximately 2.1‐fold reductions (Figure 4E). Given the central role of eDNA in matrix cross‐linking, intercellular cohesion, and stabilization of biofilm architecture, this depletion indicates significant compromise of the structural scaffold of the biofilm. Collectively, these findings demonstrate that 6‐Aminoflavone eradicates mature *K. pneumoniae* biofilms through targeted destabilization of the extracellular matrix, characterized by extensive EPS depletion and reduction of eDNA, ultimately leading to collapse of biofilm structural integrity.

### 3.7. CLSM Reveals Severe Disruption of *K*. *pneumoniae* Biofilm Architecture Following 6‐Aminoflavone Treatment

**FIGURE 5:**
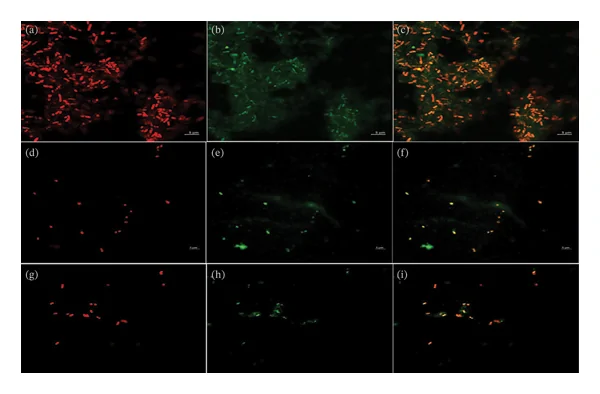
CLSM analysis of extracellular matrix components in *Klebsiella pneumoniae* biofilms following 6‐Aminoflavone treatment. Biofilms were stained with propidium iodide (PI) for extracellular DNA (eDNA) and TRITC‐Concanavalin A for exopolysaccharides. (A–C) Untreated control showed intact and dense biofilm matrix. (D–F) 1/2 MIC treatment showing noticeable reduction in eDNA and exopolysaccharide signals. (G–I) MIC treatment showed severe disruption of the biofilm matrix with markedly diminished fluorescence signals.

CLSM was performed to examine structural and compositional alterations in *K*. *pneumoniae* biofilms following 6‐Aminoflavone treatment. Biofilms were dual‐stained with TRITC‐conjugated ConA to visualize exopolysacchairdes and PI to detect eDNA—two major constituents of the EPS matrix. In untreated controls, CLSM imaging revealed dense, multilayered, and well‐organized three‐dimensional biofilm architecture, accompanied by strong ConA and PI fluorescence, indicating abundant polysaccharide and eDNA within a mature EPS matrix (Figures 5A, 5B, and 5C). In contrast, 6‐Aminoflavone–treated biofilms (1/2 MIC) displayed pronounced structural disruption, characterized by fragmented microcolonies, loss of architectural continuity, and markedly reduced fluorescence intensity for both matrix components (Figures 5D, 5E, and 5F). At the MIC, biofilm architecture was severely compromised, with CLSM images showing only sparse, loosely attached cells lacking a defined three‐dimensional structure, accompanied by a substantial reduction in both ConA and PI signals (Figures 5G, 5H, and 5I). These observations provide direct visual evidence that 6‐Aminoflavone disrupts the structural integrity of the *K. pneumoniae* biofilm matrix, leading to collapse of biofilm cohesion and destabilization of established biofilms.

**FIGURE 6:**
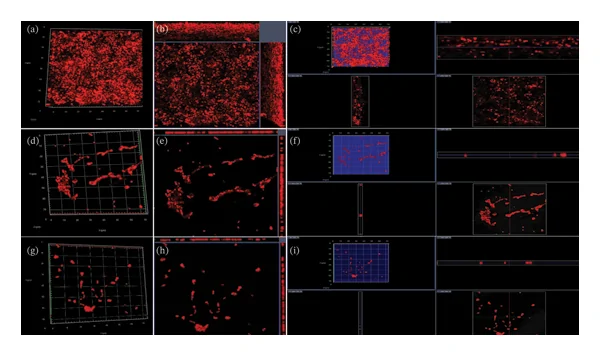
Three‐dimensional (3D) confocal laser scanning microscopy (CLSM) images demonstrating the concentration‐dependent disruption of *Klebsiella pneumoniae* biofilms by 6‐Aminoflavone. (A–C) Untreated control biofilms exhibited dense cellular aggregation and a highly organized three‐dimensional architecture. (D–F) Biofilms treated with 1/2 MIC of 6‐Aminoflavone showed disruption of biofilm integrity and reduced microcolony formation. (G–I) Biofilms treated with MIC displayed extensive architectural collapse and marked biomass depletion. The presented images are representative of three independent biological experiments.

**FIGURE 7:**
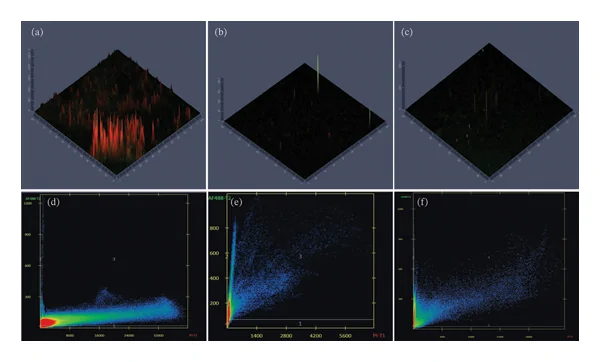
Quantitative fluorescence intensity analysis of *Klebsiella pneumoniae* biofilms following 6‐Aminoflavone treatment. (A–C) Three‐dimensional fluorescence intensity surface plots showing a concentration‐dependent reduction in fluorescence peaks and signal density in biofilms treated with 1/2 MIC (B) and MIC (C) of 6‐Aminoflavone compared with the untreated control (A), indicating progressive biofilm depletion. (D–F) Corresponding CLSM fluorescence intensity distribution plots of control (D), 1/2 MIC‐treated (E), and MIC‐treated (F) biofilms. Images shown are representative of three independent biological experiments.

Consistent with these observations, PI staining, which specifically labels the eDNA fraction of the matrix, exhibited strong red fluorescence in untreated biofilms (Figures 6A, 6B, and 6C), whereas 6‐Aminoflavone–treated biofilms (1/2 MIC and MIC) showed markedly diminished PI signals (Figures 6D, 6E, 6F, 6G, 6H, and 6I). The substantial reduction in PI fluorescence indicates significant depletion of eDNA, underscoring its critical structural role within the mature biofilm matrix. Disruption of these matrix components by 6‐Aminoflavone likely contributes to loss of biofilm integrity and enhanced biofilm eradication (Figure 7). Furthermore, degradation of eDNA‐dependent intercellular linkages likely promotes biofilm dispersal, as disruption of these cohesive interactions weakens the structural framework that maintains biofilm stability.

### 3.8. 6‐Aminoflavone Attenuates QS‐Regulated Virulence Determinants in *K*. *pneumoniae*

**FIGURE 8:**
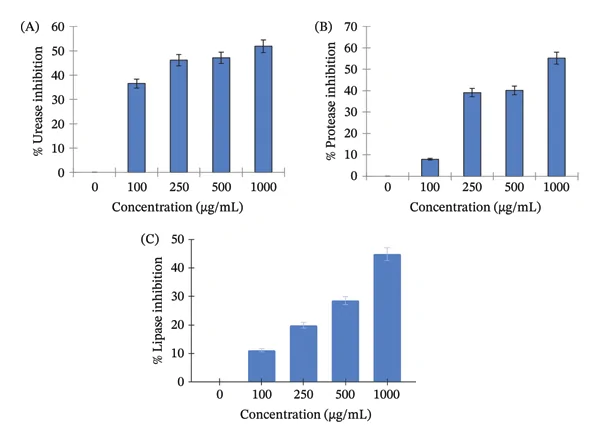
Attenuation of virulence‐associated enzyme activities in *Klebsiella pneumoniae* following treatment with 6‐Aminoflavone. (A) Urease inhibition, (B) protease inhibition, and (C) lipase inhibition. A concentration‐dependent decrease in enzyme activity is observed, indicating attenuation of virulence‐associated phenotypes. Data are presented as mean ± SD of three independent experiments (*p*  <  0.05).

The QS‐mediated antivirulence potential of 6‐Aminoflavone was evaluated by measuring the inhibition of key QS‐regulated virulence factors in *K*. *pneumoniae*, including urease, lipase, and protease activities. Treatment with 6‐Aminoflavone resulted in a significant, concentration‐dependent suppression of these virulence determinants, indicating effective interference with QS‐associated pathogenic mechanisms. Urease activity, which contributes to environmental alkalinization, bacterial persistence, and host tissue damage, showed progressive inhibition upon treatment with 6‐Aminoflavone. The enzyme activity decreased by 36.54% at 100 μg/mL, increasing to 46.15% at 250 μg/mL, 47.12% at 500 μg/mL, and reaching a maximum inhibition of 51.92% at 1000 μg/mL (Figure 8A). Protease activity, a critical virulence factor responsible for host tissue degradation and immune evasion, was also significantly suppressed following treatment with 6‐Aminoflavone. Protease inhibition increased from 7.87% at 100 μg/mL to 39.09% at 250 μg/mL, 40.07% at 500 μg/mL, and reached 55.18% at 1000 μg/mL (Figure 8B). Similarly, lipase activity, involved in lipid hydrolysis and host membrane interactions, exhibited concentration‐dependent inhibition, with 11.07%, 19.90%, 28.56%, and 44.90% inhibition observed at 100, 250, 500, and 1000 μg/mL, respectively (Figure 8C). Collectively, the coordinated inhibition of multiple QS‐regulated virulence factors demonstrates that 6‐Aminoflavone effectively attenuates QS‐mediated pathogenicity in *K*. *pneumoniae*. These findings highlight the antivirulence potential of 6‐Aminoflavone, suggesting that it suppresses bacterial pathogenic traits by targeting QS‐controlled regulatory pathways in addition to inhibiting bacterial growth.

## 4. Discussion

Antibiotic resistance has emerged as one of the most pressing global health crises of the 21st century. The WHO has identified antimicrobial resistance (AMR) as one of the top 10 global public health threats, with projections estimating up to 10 million deaths annually by 2050, if current trends persist [40, 41]. Among the ESKAPE pathogens, *K*. *pneumoniae* has gained particular notoriety as a “superbug” due to its rapid acquisition of resistance determinants, including ESBLs, carbapenemases, and colistin resistance mechanisms [42, 43]. Clinically, *K. pneumoniae* is implicated in severe nosocomial infections such as ventilator‐associated pneumonia, bloodstream infections, urinary tract infections, and device‐associated infections, particularly in immunocompromised patients [44, 45].

A defining feature of *K. pneumoniae* pathogenicity is its robust biofilm‐forming capacity. Biofilm‐associated cells exhibit up to 1000‐fold increased tolerance to antibiotics compared to their planktonic counterparts, largely due to the protective EPS matrix, altered metabolic states, and QS‐mediated regulatory networks [46–49]. QS plays a crucial role in coordinating biofilm maturation, virulence factor production, stress adaptation, and persistence [8, 50–52]. Therefore, strategies that simultaneously target bacterial growth, oxidative balance, and biofilm architecture are urgently needed.

The antibacterial activity of 6‐Aminoflavone against *K*. *pneumoniae* observed in the present study aligns with the growing interest in flavonoid‐derived compounds as alternative antimicrobial agents. In our study, 6‐Aminoflavone exhibited a concentration‐dependent bacterial growth inhibition, with an MIC of 620 μg/mL and a predominantly bacteriostatic mode of action. Although immediate bactericidal effects were not observed at lower concentrations, the compound significantly reduced clonogenic survival, indicating sustained suppression of bacterial reproductive capacity. Such bacteriostatic antimicrobial strategies are increasingly considered therapeutically valuable, particularly when acting synergistically with host immune defenses or in combination with conventional antibiotics [53, 54].

In the present study, 6‐Aminoflavone exhibited significant antibacterial activity against *K*. *pneumoniae*, characterized by concentration‐dependent suppression of the exponential growth phase (Figure 1). Similarly, earlier reports have demonstrated that quercetin and structurally related flavonoids exhibit inhibitory activity against *K*. *pneumoniae* at concentrations comparable to those observed for 6‐Aminoflavone [55, 56]. The compound primarily exerted a bacteriostatic effect, as evidenced by delayed growth kinetics and a marked decline in clonogenic survival at higher concentrations. Similar bacteriostatic antibacterial profiles have been reported for several flavonoid derivatives against Gram‐negative pathogens, where growth suppression rather than rapid lysis represents the dominant mechanism of action [10, 11].

Clonogenic survival assays further demonstrated a pronounced reduction in viable colony formation following treatment with 6‐Aminoflavone, indicating a sustained impairment of bacterial reproductive capacity (Figure 1B). Earlier studies have also reported on flavonoid‐derived antimicrobial agents that inhibit bacterial metabolic activity and replication without immediate bactericidal lysis [10]. Such bacteriostatic effects are increasingly recognized as clinically valuable, particularly when acting synergistically with host immune responses or conventional antibiotics [53].

Mechanistic investigations revealed that 6‐Aminoflavone disrupted bacterial redox homeostasis, as evidenced by elevated intracellular ROS levels and enhanced susceptibility to hydrogen peroxide–induced oxidative stress (Figure 2). These findings align with previous reports demonstrating that ROS‐mediated oxidative stress represents a common downstream mechanism of antimicrobial action, leading to DNA damage, lipid peroxidation, and protein oxidation in bacterial cells [57–59]. Similar oxidative stress–dependent antibacterial mechanisms have been documented for several plant‐derived flavonoids and phenolic compounds that generate intracellular ROS and compromise bacterial antioxidant defenses [11, 56].

Flow cytometric analysis further revealed apoptosis‐like cell death in in 6‐aminoflavone ‐treated treated *K. pneumoniae* populations (Figure 3), including membrane asymmetry disruption and progressive loss of viability, linking ROS accumulation to progressive membrane damage and loss of bacterial viability. Comparable apoptosis‐like bacterial death pathways have been described in Gram‐negative bacteria exposed to antimicrobial agents, where ROS accumulation triggers regulated cell death mechanisms rather than direct lytic destruction [59, 60]. These findings suggest that 6‐Aminoflavone induces a regulated oxidative stress–dependent cell death pathway, contributing to bacterial growth inhibition.

In addition to inhibiting planktonic growth, 6‐Aminoflavone exhibited antibiofilm activity against *K*. *pneumoniae*. Biofilm formation was inhibited by up to ∼86%, demonstrating potent suppression of early biofilm development (Figure 4A). This inhibition was accompanied by a significant reduction in CSH, a key physicochemical determinant governing bacterial adhesion to surfaces and initial microcolony formation (Figure 4B). Reduced hydrophobic interactions likely impair the ability of bacterial cells to establish stable attachment to abiotic surfaces, thereby limiting subsequent biofilm maturation. Similar relationships between decreased CSH and impaired biofilm formation have been reported in several Gram‐negative pathogens, where alterations in membrane composition or surface properties disrupt early adhesion processes [61, 62]. Thus, the observed reduction in hydrophobicity in the present study likely contributes directly to the biofilm inhibitory activity of 6‐Aminoflavone.

Beyond preventing biofilm formation, 6‐Aminoflavone also demonstrated a substantial capacity to eradicate preestablished biofilms, achieving over 80% disruption of mature biofilm biomass at higher concentrations (Figure 4C). Mature biofilms are notoriously difficult to eliminate due to the protective EPS matrix, which acts as a structural scaffold and barrier against antimicrobial agents [2, 63]. In the present study, quantitative EPS analysis revealed a dramatic depletion of extracellular polysaccharides following treatment, indicating severe compromise of matrix integrity (Figure 4D). In parallel, eDNA—another key structural component involved in matrix cross‐linking and stabilization—was also significantly reduced in treated biofilms (Figure 4E). The combined reduction of EPS and eDNA suggests that 6‐Aminoflavone disrupts multiple structural elements of the biofilm matrix, thereby weakening intercellular cohesion and promoting biofilm disassembly. These observations were further validated by CLSM, which revealed marked architectural collapse of biofilm structures, accompanied by a pronounced reduction in fluorescence signals corresponding to both polysaccharides and eDNA (Figures 5, 6, and 7).

In addition to matrix disruption, 6‐Aminoflavone significantly attenuated QS‐regulated virulence factors, including urease, lipase, and protease activities. These enzymes play critical roles in bacterial pathogenicity by facilitating nutrient acquisition, host tissue degradation, and biofilm development [8, 50, 52, 64]. QS systems function by coordinating gene expression in a cell density–dependent manner, particularly regulating phenotypes such as biofilm formation and secretion of virulence‐associated factors, including degradative enzymes. The coordinated suppression of these QS‐regulated determinants indicates that 6‐Aminoflavone interferes with bacterial communication networks that regulate virulence expression and biofilm maturation (Figure 8). Earlier studies have also demonstrated that several plant‐derived polyphenols can attenuate QS‐regulated virulence in bacterial pathogens. Compounds such as quercetin, ferulic acid, gallic acid, *p*‐coumaric acid, and syringic acid have been reported to suppress QS‐controlled phenotypes—including biofilm formation, motility, and extracellular enzyme production—in pathogens such as *Pseudomonas aeruginosa*, *Staphylococcus aureus*, *K*. *pneumoniae*, and *Staphylococcus epidermidis*. These findings indicate that polyphenolic molecules can interfere with bacterial communication networks that regulate virulence expression and biofilm development, thereby reducing pathogenic potential without directly exerting strong bactericidal pressure [65–73].

Taken together, the present study demonstrates that 6‐Aminoflavone exerts potent antibacterial and antibiofilm activity against *K*. *pneumoniae* through interconnected mechanisms involving oxidative stress induction, apoptosis‐like bacterial death, extracellular matrix destabilization, and attenuation of QS‐regulated virulence. Mechanistically, 6‐Aminoflavone induced substantial intracellular ROS accumulation and enhanced oxidative stress susceptibility, indicating disruption of bacterial redox homeostasis. Excessive ROS generation is known to impair membrane integrity and trigger apoptosis‐like bacterial death pathways [57–59], consistent with the apoptosis‐like phenotypes observed in the present study. In parallel, 6‐Aminoflavone significantly inhibited biofilm formation and eradicated mature biofilms through depletion of EPS, reduction in eDNA, attenuation of CSH, and collapse of biofilm architecture. Previous studies have similarly reported that flavonoid derivatives induce ROS‐mediated disruption of biofilm‐associated pathways and virulence regulation in bacterial pathogens [12, 14, 56]. Furthermore, significant suppression of QS‐regulated virulence factors, including urease, protease, and lipase, indicates interference with bacterial communication networks governing pathogenicity and biofilm maturation [8, 50]. Collectively, these findings support a mechanistic framework in which ROS‐mediated oxidative imbalance contributes to bacterial growth inhibition, matrix destabilization, biofilm disruption, and attenuation of virulence in *K. pneumoniae.* Nevertheless, further studies employing ROS scavengers and molecular analyses will be necessary to establish direct mechanistic causality.

## Author Contributions

Pooja Pandey carried out the antibiofilm experiments, verified the findings, and prepared the initial draft of the manuscript. Tushar performed the antibacterial studies and contributed to data analysis, visualization, methodology development, data curation, and manuscript writing. Dr. Lakshmi Sirisha Vavilala oversaw the study and contributed to conceptualization, experimental design, validation, data interpretation, visualization, project administration, funding acquisition, and manuscript development.

## Funding

This research did not receive any specific grant from funding agencies in the public, commercial, or not‐for‐profit sectors.

## Disclosure

All authors critically reviewed and approved the final version of the manuscript and are accountable for the accuracy and integrity of the work.

## Conflicts of Interest

The authors declare no conflicts of interest.

## Data Availability

The datasets generated and analyzed during the current study are available from the corresponding author upon reasonable request.
